# The Determination of Immunomodulation and Its Impact on Survival of Rectal Cancer Patients Depends on the Area Comprising a Tissue Microarray

**DOI:** 10.3390/cancers12030563

**Published:** 2020-02-29

**Authors:** Elisabeth S. Gruber, Georg Oberhuber, Dietmar Pils, Theresa Stork, Katharina Sinn, Sylvia Gruber, Robert Nica, Dan Kolmer, Suzanne D. Turner, Michaela Schlederer, Joachim Widder, Wolfgang Doerr, Béla Teleky, Lukas Kenner

**Affiliations:** 1 Division of General Surgery, Department of Surgery, Medical University of Vienna, 1090 Vienna, Austria; elisabeth.s.gruber@meduniwien.ac.at (E.S.G.); bela.teleky@meduniwien.ac.at (B.T.); 2 Comprehensive Cancer Center, Medical University of Vienna, 1090 Vienna, Austria; oberhuber@patho.at (G.O.); dietmar.pils@meduniwien.ac.at (D.P.); t.stork@gmx.at (T.S.); katharina.sinn@gmail.com (K.S.); sylvia.gruber@meduniwien.ac.at (S.G.); michaela.schlederer@meduniwien.ac.at (M.S.); joachim.widder@meduniwien.ac.at (J.W.); wolfgang.doerr@meduniwien.ac.at (W.D.); 3 Department of Experimental and Translational Pathology, Clinical Institute of Pathology, Medical University of Vienna, 1090 Vienna, Austria; 4 INNPATH GmbH, Tyrol Clinics, Innsbruck, 6020 Innsbruck, Tyrol, Austria; 5 Department of Surgery, Medical University of Vienna, 1090 Vienna, Austria; 6 Department of Radiation Oncology, Christian Doppler Laboratory for Medical Radiation Research for Radiation Oncology, Comprehensive Cancer Center, Medical University of Vienna, 1090 Vienna, Austria; 7 TissueGnostics Austria Global Headquarter, TissueGnostics GmbH Vienna, 1020 Vienna, Austria; robert.nica@tissuegnostics.com (R.N.); dan.kolmer@tissuegnostics.com (D.K.); 8 Department of Pathology, University of Cambridge, Cambridge CB2 1QP, UK; sdt36@cam.ac.uk; 9 Central European Institute of Technology, Masaryk University, 602 00 Brno, Czech Republic; 10 Christian Doppler Laboratory for Applied Metabolomics (CDL-AM), Division of Nuclear Medicine, Medical University of Vienna, 1090 Vienna, Austria; 11 Unit of Laboratory Animal Pathology, University of Veterinary Medicine Vienna, 1210 Vienna, Austria; 12 CBmed Vienna, Medical University of Vienna, 1090 Vienna, Austria

**Keywords:** Irradiated rectal cancer, Immunoscore, virtual microscopy, digital pathology, tissue microarray (TMA)

## Abstract

Background: T cell density in colorectal cancer (CRC) has proven to be of high prognostic importance. Here, we evaluated the influence of a hyperfractionated preoperative short-term radiation protocol (25 Gy) on immune cell density in tumor samples of rectal cancer (RC) patients and on patient survival. In addition, we assessed spatial tumor heterogeneity by comparison of analogue T cell quantification on full tissue sections with digital T cell quantification on a virtually established tissue microarray (TMA). Methods: A total of 75 RC patients (60 irradiated, 15 treatment-naïve) were defined for retrospective analysis. RC samples were processed for immunohistochemistry (CD3, CD8, PD-1, PD-L1). Analogue (score 0–3) as well as digital quantification (TMA: 2 cores vs. 6 cores, mean T cell count) of marker expression in 2 areas (central tumor, CT; invasive margin, IM) was performed. Survival was estimated on the basis of analogue as well as digital marker densities calculated from 2 cores (Immunoscore: CD3/CD8 ratio) and 6 cores per tumor area. Results: Irradiated RC samples showed a significant decrease in CD3 and CD8 positive T cells, independent of quantification mode. T cell densities of 6 virtual cores approximated to T cell densities of full tissue sections, independent of individual core density or location. Survival analysis based on full tissue section quantification demonstrated that CD3 and CD8 positive T cells as well as PD-1 positive tumor infiltrating leucocytes (TILs) in the CT and the IM had a significant impact on disease-free survival (DFS) as well as overall survival (OS). In addition, CD3 and CD8 positive T cells as well as PD-1 positive TILs in the IM proved as independent prognostic factors for DFS and OS; in the CT, PD-1 positive TILs predicted DFS and CD3 and CD8 positive T cells as well as PD-1 positive TILs predicted OS. Survival analysis based on virtual TMA showed no impact on DFS or OS. Conclusion: Spatial tumor heterogeneity might result in inadequate quantification of immune marker expression; however, if using a TMA, 6 cores per tumor area and patient sample represent comparable amounts of T cell densities to those quantified on full tissue sections. Consistently, the tissue area used for immune marker quantification represents a crucial factor for the evaluation of prognostic and predictive biomarker potential.

## 1. Introduction

A balanced tumor microenvironment can ignite tumor immune surveillance to promote local and distant tumor control [1,2,3,4,5,6,7]. The Immunoscore, that is based on the tumor immune contexture, has recently been validated as a prognostic factor for survival of colorectal cancer (CRC) patients [8]. The clinical implementation of immune checkpoint blockade has emerged as one of the greatest advantages in cancer immunotherapy and can lead to long-term regression [9,10]. An “immune-high” tumor microenvironment and the consecutive activation of the immune checkpoint axis is ascribed to CRCs with a high mutational load (15% microsatellite unstable tumors) [11,12]. Accordingly, immune checkpoint blockade is associated with improved survival in this subgroup of patients; however, the majority of patients present with ”immune-low” tumors that are not responsive to immune checkpoint blockade [13].

Irradiation can ignite pro-immunogenic effects, that depend on dose, fractionation and sequencing [14]. If appropriately applied, it might shape a tumor microenvironment potentially responsive to immune checkpoint blockade. However, data on synergistic effects of irradiation and immunotherapy are inconsistent, since irradiation protocols that have been investigated were chosen empirically rather than rationally [15]. In rectal cancer (RC) specimens, preoperative radiochemotherapy resulted in the significant infiltration of CD8 positive T cells whereas the expression of immune checkpoint receptors remained stable [16]. Good histopathological response to preoperative treatment was associated with CD3 and CD8 positive T cell densities [16,17,18]. In a murine CRC model, ablative irradiation led to an increase in CD8 positive lymphocytes in the tumor microenvironment, whereas fractionated irradiation showed lower levels of lymphocyte infiltration [19]. However, recent reports have investigated different preoperative treatment protocols that demonstrate heterogeneous data on tumor immune cell infiltration quantified with different methods (biopsies, tissue microarray (TMA), full tissue sections).

Recently, several issues have been raised concerning the method of immune marker quantification employed. Spatial tumor heterogeneity of target molecules in cancer tissue is well described [20,21,22,23,24,25,26]; however, histologic sampling by TMA is still regularly used for marker analysis [18,27,28], although it involves the risk of underestimation [22,24,28]. The Immunoscore uses two cores in two tumor areas each (CT, IM) for quantification of immune cell densities in CRC [18,27]. Interestingly, heterogeneity of tumor infiltrating immune cell densities in primary CRCs compared to intralesional homogeneity of infiltrating immune cells in hepatic metastases has been confirmed in a recent study by Halama et al.; here, the evaluation of single fields for immune cell density in hepatic metastases showed that values in up to five fields did not add further approximation to the average immune cell value [29].

In this study, we evaluated an immunomodulatory effect of a hyper-fractionated short-term radiation protocol in RC patients and the proposed differences in immune marker densities that might be caused by the choice of mode of analysis using full tissue sections versus (virtual) TMA; in addition, we defined the minimal tissue area needed for quantification. The results of this study should contribute to the body of knowledge on irradiation-induced immunogenicity by elucidating the immunogenic effects of a clinically employed preoperative radiation protocol [30]; furthermore, these data should encourage the optimization of effective and tissue-saving immune cell marker quantification.

## 2. Results

### 2.1. Preoperative Radiation Therapy is Associated with a Lower Density of Infiltrating T Cells and a Lower Dukes Stage with High Levels of PD-L1 Positive Tumor Infiltrating Lymphocytes (TILs)

Quantification of infiltrating T cells (cells positive for CD3 or CD8 expression) on full tumor tissue sections, showed that preoperative radiation therapy was associated with a significant reduction in CD8 positive T cell density in the central tumor (CD8: *p =* 0.017) as well as reduced CD3, CD8 density and PD-1 expression level on TILs at the invasive margin (CD3: *p =* 0.006, CD8: *p =* 0.001, PD-1: *p =* 0.005). In addition, high PD-L1 expression in the central tumor as well as at the invasive margin correlated with a lower Dukes stage (PD-L1: CT: R = –0.23, *p =* 0.046; IM: R= –0.24, *p =* 0.041; Table 1, Table 2, Table 3 and Table 4). Of note, PD-1 and PD-L1 expression were exclusively detected on TILs, but not on tumor cells.

### 2.2. A decrease in T cell density is observed only at the invasive margin of tumors that have been exposed to radiation therapy

**Figure 1:**
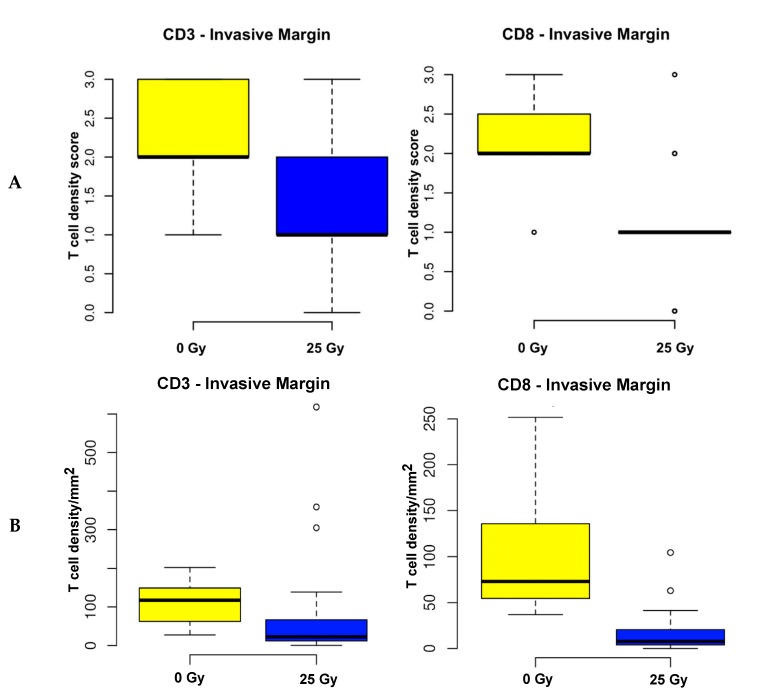

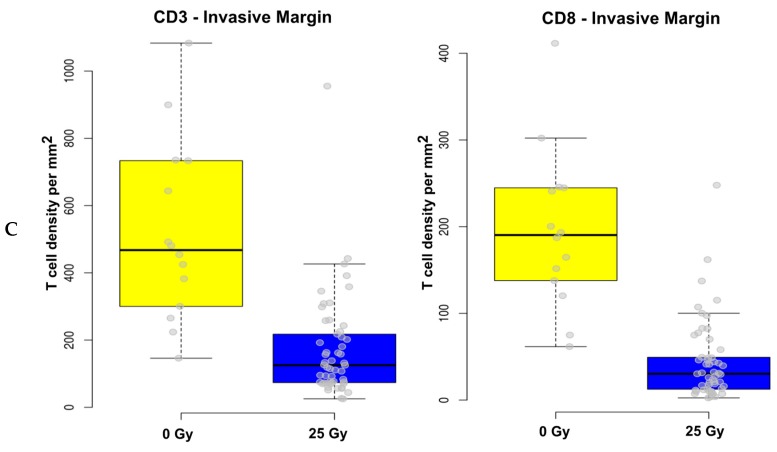
Correlative analysis of T cell density in preoperatively irradiated vs. treatment-naïve rectal cancer specimens quantified by analogue using full tissue sections (**A**) and on virtually established tissue microarray (TMA; (**B**) 2 cores per tumor region and (**C**) 6 cores per tumor region). **A.** 25 Gy of preoperative irradiation significantly attenuates marginal density of CD8 positive T cells observed on full tissue sections (*p <* 0.0001); a trend was shown towards less CD3 density in the IM (*p =* 0.0970). **B.** 25 Gy of preoperative irradiation significantly attenuates marginal density of CD3 as well as CD8 positive T cells seen on virtual TMAs using 2 cores (*p <* 0.0001 each). **C**. 25 Gy of preoperative irradiation significantly attenuates marginal density of CD3 as well as CD8 T cells seen on virtual TMA using 6 cores (*p <* 0.0001 each).

To validate these exploratory findings, the full patient cohort (60 irradiated and 15 treatment-naïve RC samples) were analyzed following immunohistochemical staining of full tissue sections. T cell and immune checkpoint protein expression were compared according to their staining intensity. Less CD8 density and a trend towards less CD3 density was observed at the invasive margin of full tissue sections (*p <* 0.0001 and *p =* 0.0970; Figure 1A); no significant differences were found in CD3 and CD8 density in the central tumor (*p =* 1 and *p =* 0.183, respectively).

**Figure 2:**
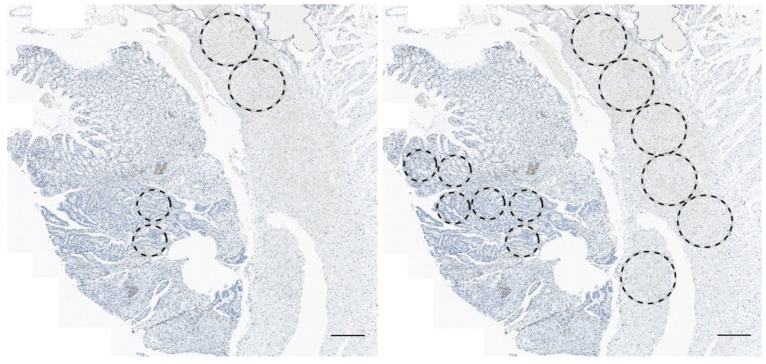
Virtual TMA establishment on scanned full section slides of rectal cancer (RC) samples using StrataQuestTM^®^. (**Left**): Randomly selected virtual cores in the central tumor (2x 0.6mm diameter) and in the invasive margin (2x 1mm diameter) on full section slides of RC samples. (**Right**)**:** Expansion of virtual core establishment from 2 to a maximum of 6 cores per tumor region taking into account the minimum amount of tumor area available amongst all full section slides. **Note**. Bar graph = 500 µm.

In order to determine whether analysis of sample cores as opposed to full tissue sections provides similar results therefore negating the need to analyze full tissue sections, a virtual TMA was established using two cores from each tumor area of the scanned full section (Figure 2, left). Analysis of irradiated vs. treatment-naïve RC samples applying this methodology showed significantly less CD3 and CD8 T cell density in irradiated RC samples at the invasive margin (*p <* 0.0001 each, Figure 1B); again, no significant differences were found between CD3 and CD8 density in the central tumor (*p =* 0.864 and *p =* 0.227, respectively). Similarly, analysis of irradiated vs. treatment-naïve RC samples using 6 cores from each tumor area showed significantly less CD3 and CD8 T cell density in irradiated RC samples at the invasive margin (*p <* 0.0001 each, Figure 1C); no significant differences were found between CD3 and CD8 density in the central tumor (*p =* 0.202 and *p =* 0.424, respectively). These data are suggestive of immunosuppression in irradiated RC samples. Of note, analogue quantification of T cell density and immune checkpoint protein expression demonstrated a heterogeneous pattern of immune cell clusters spread across the tumors (Supplementary Figure S1).

### 2.3. T Cell Density Correlates between Quantification Methods

**Figure 3:**
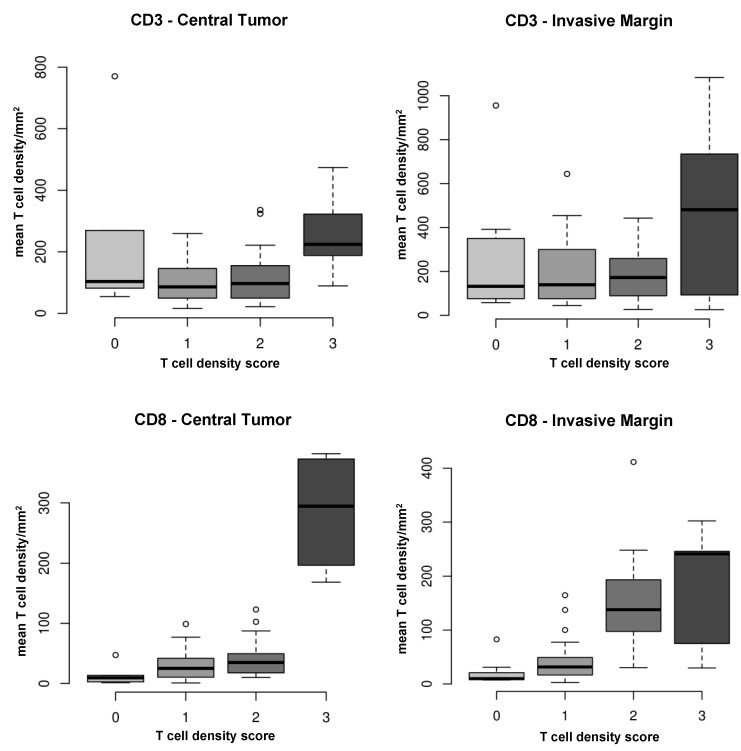
Correlative analysis of T cell densities quantified on full tissue sections (score 0–3) vs. mean T cell densities of 6 cores. T cell densities of the 2 quantification methods match with respect to correlational strength in the different tumor areas. **Top**: Tumoral and marginal CD3 positive T cells: R = 0.35 (medium correlation) and R = 0.14 (weak correlation), respectively. **Bottom:** Tumoral and marginal CD8 positive T cells: R = 0.45 (medium correlation) and R = 0.69 (good correlation), respectively. **Note.** Score: no (0), low (1), moderate (2) or high (3) protein levels with respect to positive T cells or TILs present; R—Spearman’s correlation coefficient.

Due to the improved significance of T cell density in the invasive margin when quantified on a virtual TMA (compared to full tissue sections), we expanded the number of virtual cores to a maximum of six per tumor area, Figure 2, right). We then compared full section scores with mean T cell densities of the core 1–6. For the most part, T cell density scores on full tissue sections corresponded with mean T cell density determined on the virtual TMA. However, results showed positive, but variable correlations between quantification methods in each tumor region, with weak correlations between CD3 density (tumoral and marginal CD3 positive T cells: R = 0.35 and R = 0.14, respectively) and moderate to strong correlations in CD8 density (tumoral and marginal CD8 positive T cells: R = 0.45 and R = 0.69, respectively; Figure 3).

### 2.4. Determination of T Cell Density Varies According to the Number of Cores Comprising the Virtual TMA

**Figure 4:**
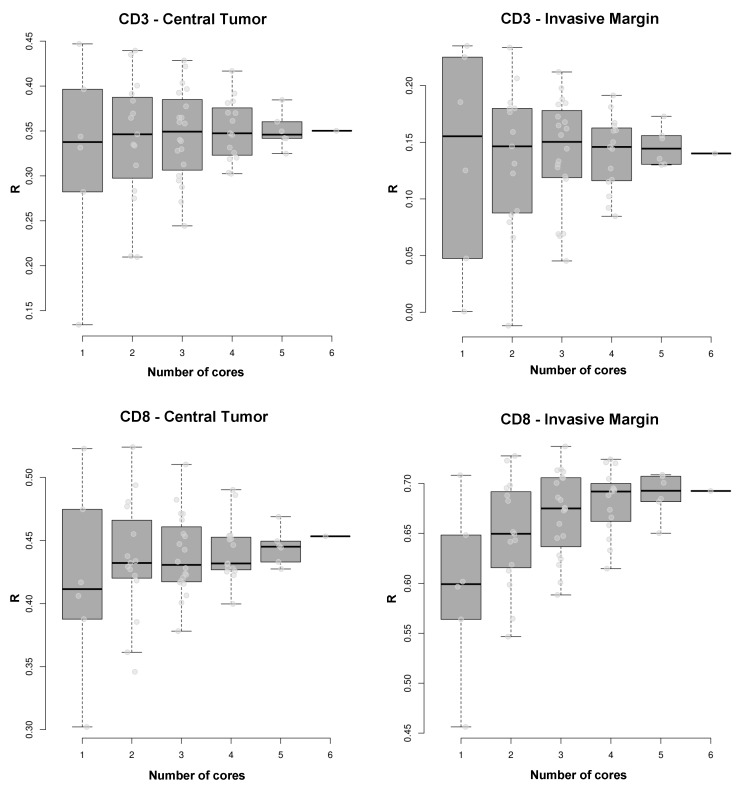
Variance of correlations between T cell densities using analogue quantification and T cell densities using any possible combination of 1 to 6 virtual TMA cores to estimate the mean. Ascending numbers of cores resulted in a more precise approximation of correlation to the analogue values in each tumor area, except for marginal CD3 density. **Top**: Tumoral CD3: 1 core, R = 0.34 vs. 6 cores, R = 0.35 (moderate correlation); marginal CD3: 1 core, R = 0.16 vs. 6 cores, R = 0.14 (weak correlation), respectively. **Bottom**: Tumoral CD8: 1 core, R = 0.41 vs. 6 cores, R = 0.46 (strong correlation); marginal CD8: 1 core, R = 0.60 vs. 6 cores, R = 0.69 (strong correlation), respectively; however, adding cores for quantification analysis led to a more precise approximation of the correlation between analogue and virtual TMA quantification. **Note.** R—Spearman’s correlation coefficient.

To test whether T cell density depends on the number of cores used for quantification of each tumor area, we analyzed the relationship between mean T cell density of all patients by correlating the mean T cell densities of six cores with the mean T cell densities determined from one up to five cores quantified by virtual TMA. Here, increasing numbers of cores demonstrated positive, but variable correlations in each tumor region: tumoral CD3: 1 core, R = 0.34 vs. 6 cores, R = 0.35 (moderate correlation); marginal CD3: 1 core, R = 0.16 vs. 6 cores, R = 0.14 (weak correlation), respectively; tumoral CD8: 1 core, R = 0.41 vs. 6 cores (moderate correlation), R = 0.46; marginal CD8: 1 core, R = 0.60 vs. 6 cores, R = 0.69 (strong correlation), respectively; however, determination of T cell density using six cores resulted in the highest correlation between quantification methods (versus 1–5 cores; Figure 4).

### 2.5. Approximation of T Cell Density between Quantification Methods Does not Depend on the Specific T Cell Density of Each Core

**Figure 5:**
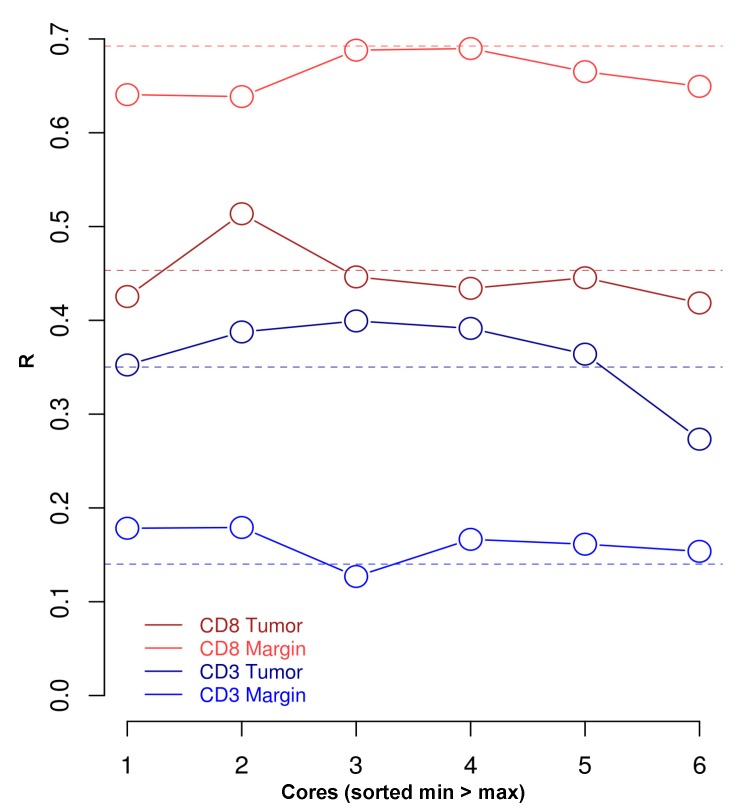
Correlation of the mean T cell densities of 6 cores with mean T cell densities of full tissue sections (dashed line) versus ascending sorted mean T cell densities of 1–6 cores. Strength of correlation between T cell densities on full tissue sections and virtual TMA does not depend on the mean T cell density of the cores chosen for correlation. **From bottom to top:** Tumoral CD3 – low T cell density core, R = 0.35 vs. high density core, R_s_ = 0.26 (moderate correlation), max. core 3, R = 0.40; marginal CD3 – low T cell density core, R = 0.18 vs. high density core, R = 0.17 (weak correlation), max. core 2, R = 0.19; tumoral CD8 – low T cell density core, R = 0.42 vs. high density core, R = 0.40 (moderate correlation), max. core 2, R = 0.51; marginal CD8 - low T cell density core, R = 0.64 vs. high density core, R = 0.66 (strong correlation); max. core 4, R = 0.70. **Note.** R—Spearman’s correlation coefficient.

To test whether the approximation of T cell densities depends on the specific cores (or core location) used for quantification and hence would result from a systematic bias, we analyzed the cores sorted in ascending T cell densities (using the core with the lowest (1) to the highest (6) mean T cell density) and compared them with the correlations between the mean T cell densities quantified by 6 cores versus mean T cell densities on full tissue sections. We show that using cores 2, 3 and 4 (comprising medium to high T cell densities) results in the strongest correlation, demonstrating that the approximation of T cell densities is not related to the actual T cell density (and location) of each individual core, which makes a systematic bias unlikely (Figure 5).

### 2.6. T Cell Density Varies Independently of the Number of Cores Chosen for Quantification by TMA

**Figure 6:**
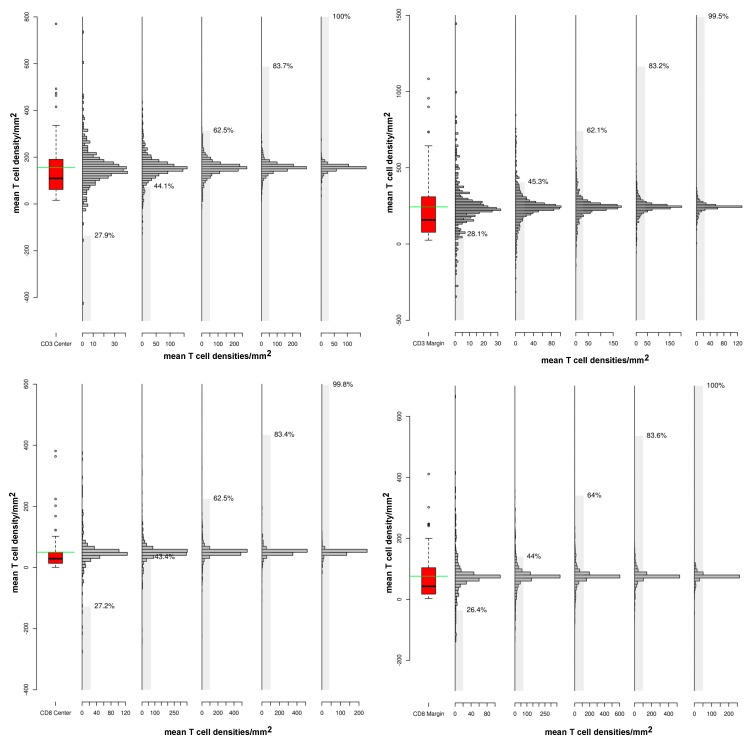
Variations in T cell densities based on the number of cores chosen for quantification by virtual tissue microarray. Quantification of mean T cell densities using 5 cores for each patient (bar graph) shows a higher range of variation compared to mean T cell densities using 6 cores from all patients (boxplot). **Top**: Tumoral CD3 density: range 0–350 (boxplot); 1 core: 0–40 (SE = 28.1%) vs. 5 cores: 0–150 (SE = 99.5%); marginal CD3 density: range 0–650 (boxplot); 1 core: 0–30 (SE = 27.9%) vs. 5 cores 0–120 (SE = 100%). **Bottom**: Marginal CD8 density: range 0–100 (boxplot); 1 core: 0–120 (SE = 27.2%) vs. 5 cores 0-200 (SE = 99.8%); tumoral CD8 density: range 0–200 (boxplot); 1 core: 0–100 (SE = 26.4%) vs. 5 cores: 0-250 (SE = 100%). **Note**. Range - range of variation in T cell density; SE—standard error.

We further wanted to evaluate how the number of cores chosen for averaging influences the variation in T cell density and how this methodological variation relates to the biological variation of all six cores. For this, the biological variation of all six cores was determined (boxplot) and the deviation to the ground truth of these six cores was calculated using all possible combinations of one to five cores for each parameter and each patient (histograms). We depicted these differences as distributions by ascending numbers of cores used for averaging parameters (one to five, grey histograms, respectively). Here, similar ranges of deviation of parameters were found compared to the respective biological variation of all six cores (boxplot), even when using all combinations of five cores of each patient. Grey bars show the percentages of averaged parameters inside the standard error (SE) of the six core values for each patient (Figure 6). However, the results of five averaged cores are not statistically different to the results from six cores, as (almost) all values (99.5%–100%) are inside the standard error range; in fact, by using only four cores almost 20% of combinations are outside the SE range.

### 2.7. Survival Analysis

The median follow-up was 10.5 years (CI_95_ 7.4–13.2) for all 75 patients, 12.6 years (CI_95_ 10.5–14.9) for the group that received radiation therapy and 7.2 years (CI_95_ 3.9–8.2) for the treatment-naïve group. During this time, 61 patients died; of these, 47 patients had received radiation therapy preoperatively and 13 patients had not. With regard to DFS, 20 patients relapsed after curative surgery; of these, 17 patients had received radiation therapy preoperatively and 3 patients had not. With regard to OS, out of 75 patients comprising the entire cohort, 14 patients died after curative surgery; of these, 13 patients had received radiation therapy preoperatively and 1 patient had not.

### 2.8. Analysis of Disease-free and Overall Survival based on T Cell Densities and Immune Checkpoint Protein Expression by Analogue Quantification on Full Tissue Sections

Cox Regression analysis based on T cell densities and immune checkpoint protein expression on TILs quantified on full tissue sections was performed for DFS and OS. Here, CD3 density and PD-1 expression in both tumor areas (CT, IM) showed a significant impact on both DFS and OS (CD3: CT, *p =* 0.018/IM, *p =* 0.002 and PD-1: CT, *p =* 0.037/IM, *p =* 0.016, respectively), whilst PD-L1 expression showed a trend towards improved survival (PD-L1: CT, *p =* 0.087/IM, *p =* 0.078); with regard to CD8 density, only expression in the IM was associated with improved survival. Radiation therapy showed no impact, whilst patients with nodal positive RC had a 3.69 chance of relapse or death (*p =* 0.020).

In a multiple Cox Regression model, nodal status remained as a crucial prognostic factor for DFS next to T cell density and immune checkpoint protein expression on TILs, whilst radiation therapy did not; however, CD3 density in the IM (*p =* 0.004) as well as PD-1 expression in the IM (*p =* 0.045) and the CT (*p =* 0.048) was shown to be an independent prognostic marker for DFS taking into account nodal status and preoperative radiation therapy as crucial factors influencing survival; CD8 density (*p =* 0.074) in the IM as well as PD-L1 expression in the IM and the CT (*p =* 0.067 and *p =* 0.050, respectively) showed a trend towards impaired DFS next to nodal status (Table 5). In a multiple Cox Regression model for OS, again nodal status remained a crucial prognostic factor next to T cell density or immune checkpoint protein expression on TILs, whilst radiation therapy did not; however, all factors tested are independent prognostic markers for OS (CD3: CT, *p =* 0.021/IM *p =* 0.002; CD8: CT, *p =* 0.029/IM, *p =* 0.029; PD-1: CT *p =* 0.037/IM *p =* 0.017) taking into account the nodal status and preoperative radiation therapy as crucial factors influencing survival; PD-L1 expression in the IM showed a trend towards influencing survival, whilst PD-L1 expression in the CT did not (Table 6).

### 2.9. Analysis of Disease-free and Overall Survival based on the Immunoscore Quantified on Virtual TMAs

In contrast to the prognostic impact of T cell densities quantified on full tissue sections, the Immunoscore (2 cores in the CT, 2 cores in the IM) showed no impact on DFS or OS by univariate analysis and the multiple model (next to nodal status and preoperative radiation therapy) in this cohort of patients (Table 7 and Table 8).

### 2.10. Analysis of disease-free and overall survival based on 6 cores per tumor area quantified on virtual TMAs

Here, tumoral CD3 density was the only factor with a trend towards an impact on DFS and with independent prognostic power for DFS (CD3: CT, *p =* 0.093 and *p =* 0.093, respectively). All other factors remained insignificant (Table 9). With regard to OS, tumoral CD3 density and nodal status showed a trend towards an impact on IS and an independent prognostic power for OS (CD3: CT, *p =* 0.052 and *p =* 0.084, respectively); in addition, nodal status as a factor in the multiple model tended to remain an independent prognostic marker of OS next to T cell density and preoperative radiation therapy (nodal status, *p =* 0.074; Table 10).

## 3. Discussion

The impact of the tumor immune contexture on the prognosis of CRC patients is not doubted and the consensus Immunoscore is internationally validated for implementation of a “TNM-Immune classification” of cancer [31]. However, concerns have been raised regarding the methodology used for immune marker quantification using TMA in particular [20,21,22,23,24,25,32].

In this study, we primarily aimed to explore the impact of a hyperfractionated short-term preoperative radiation protocol on immunomodulation; hereby, we have been confronted with the issue of spatial tumor heterogeneity, which led us to investigate different methods of quantifying immune cells on virtual TMA versus full tissue sections in a structured approach. We found that hyperfractionated short-term preoperative radiation therapy resulted in a decrease in immune cell infiltration and that the tissue area represents a crucial factor in assessing immune markers: If quantified on full tissue sections, CD3 positive, CD8 positive T cells and PD-1 positive TILs had a significant impact and represent independent prognostic factors for DFS and OS, whilst survival analysis based on virtual TMA remained insignificant.

Irradiation promotes pro-immunogenic effects, that depend on dose, fractionation and sequencing. Recent studies report that irradiation doses in low ranges promote an innate and adaptive immune response that supports building anti-tumor immunity [14,33,34]. We here evaluated tumor immune infiltration after treatment with a hyperfractionated short-term radiation protocol, that involves a lower dose than usual (cumulative dose 25 Gy: 2× 2.5 Gy over 5 days), which still resulted in an overall decrease of immune marker expression (T cells, immune checkpoint proteins) compared to treatment-naïve RC samples. The infiltration of stromal CD8 positive T cells has been demonstrated to double in samples of RC patients treated with preoperative chemoradiotherapy (cumulative dose 20 Gy: 1× 4 Gy over 7 days with concomitant administration of tegafur/uracil) [35]. Another study using the same methodology, investigated T cell infiltration and immune checkpoint protein expression after extensive chemoradiation (cumulative dose 40–45 Gy: 25-28 fractions over 5 weeks with concomitant fluoropyrimidine-based chemotherapy) and found an increase in CD8 positive T cells and only very low PD-L1 protein expression on stromal cells; similar to our study, CD8 positive T cells were associated with a favorable outcome [16]. In both studies, T cell infiltration and immune checkpoint protein expression was quantified analoguously comparing pretreatment biopsies and posttreatment resected full tissue sections.

With regard to immune checkpoint proteins, quantification is challenging due to weak staining intensity depending on the antibody used [22,28]. Accordingly, recent reports investigating different treatment protocols showed conflicting data on post-treatment immune checkpoint protein expression in RC [36,37,38,39]; of note, a meta-analysis demonstrated general agreement on the association of PD-L1 protein expression with poor prognosis and on its applicability as a biomarker in CRC patients [40]. Our data, based on full section analysis, support the fact that patients with advanced RC (Dukes stage D) demonstrate lower infiltration of PD-L1 positive TILs both in the CT and the IM. In addition, we found that expression of PD-1 positive TILs (and tendentially PD-L1) in the CT and the IM was associated with poor prognosis and proved as an independent prognostic factor for DFS and OS. These findings might be explained by tumor immune evasion that results from T cell anergy upon interaction of the inhibitory immune checkpoint protein PD-1 with PD-L1 expressed on tumor cells or antigen-presenting cells.

Upon quantification of full sections for immune marker expression, we encountered the issue of spatial tumor heterogeneity that is well described in cancer tissue [20,21,22,23,24,25,26]. Heterogeneous clustering of CD3 and CD8 positive T cells, as well as PD-1 and PD-L1 positive TILs was evident; however, evaluation by board-certified pathologists ensured that these heterogeneous expression patterns were seen in the context of the entire tumor section, whilst digital quantification on selected tissue cores would not have allowed any conclusions to be drawn regarding the spatial structure of the tumor. Consistently, the irradiation-induced immune ablation we found here is controversial to the reports using biopsies for quantification [16,35]. Hence, TMA-based marker analysis might result in incorrect cut-off definitions for tools like the Immunoscore on which prognostic decisions are based on [18,27]. Still TMAs are used routinely as they offer cost effective analysis of large patient cohorts.

However, since this is an outstanding case that conflicts the above-mentioned data on immunomodulation after (chemo)radiotherapy, we focused on reconstruction of the initial scenario by virtually establishing TMA cores for digital quantification. We found that T cell densities correlated using both quantification methods; lower correlations in CD3 densities might be ascribed to the lower staining intensity of the antibody used. However, a trained and validated algorithm was used for digital analysis and each slide was manually corrected by a trained observer. We therefore decided to expand virtual TMA core establishment to evaluate whether the tissue area (represented by the number of cores) used for quantification influenced the approximation of T cell density. As previously described, expanding the area for quantification using bigger cores in the case of a TMA or upgrading to full section analysis minimizes the chance of measuring heterogeneous marker patterns [22,24,28]. Hence, correlations between analogue and digital estimations were improved by increasing the number of TMA cores used for quantification of T cell densities, independent of the mean T cell count of the core (and hence location) chosen for quantification. In fact, T cell density varied largely between the TMA cores, which once more might be attributable to spatial tumor heterogeneity.

These findings were reflected in the survival analysis conducted using immune cell marker density quantified on full tissue sections versus virtual TMAs. Similar to recent reports, survival analysis based on full section quantification demonstrated a significant prognostic impact for high CD3 and CD8 positive T cell (as well as PD-1 positive TIL) densities in the CT and the IM on DFS as well as OS. Neither survival analysis based on virtual TMA nor the Immunoscore showed prognostic significance, when analyzed using two cores for each tumor area (CT, IM) as previously described in RC patients [18]. Since the Immunoscore has recently been internationally validated and the impact of tumor immune cell infiltration on prognosis of CRC patients is not doubted [31], we attribute the insignificant results to the small number of patients (n = 75) comprising this study cohort.

In addition to the small sample size, another limitation of this study is that the area of most RC samples was relatively small and therefore virtual TMA establishment for digital quantification was limited to 6 cores per tumor area. However, we demonstrate here that 6 cores per tumor area represent comparable T cell densities as those quantified on full tissue sections and an extension of the area for digital evaluation by virtual TMA would therefore have been redundant.

## 4. Materials and Methods

### 4.1. Study Cohort

A total of 75 patients with RC that were subsequently operated on between 2000 and 2009 at the Medical University of Vienna were selected and retrospectively analyzed. Out of these, 60 patients had locally advanced RC and were treated after a hyper-fractionated short-term pre-operative radiation therapy protocol [30]. Within this protocol, patients received 2× 2.5 Gy applied twice daily over 5 days (Monday to Friday) to a total dose of 25 Gy. Surgery was performed the week thereafter. Partial and total mesorectal excision with a stapled anastomosis for tumors located in the upper third and the middle and lower rectum was performed, respectively. Protective ileostomies were added at the surgeon’s discretion. Another 15 treatment-naïve human RC samples were collected as a control population. Each sample was selected by a board-certified pathologist. Patient data were handled according to Good Scientific Practice (GSP) Guidelines. Radiographic data were analyzed according to the response evaluation criteria in solid tumors (RECIST). The study was approved by the ethics committee of the Medical University of Vienna (EK #1197/2019).

### 4.2. Immunohistochemistry (IHC)

IHC was conducted on 1 µm thick full tissue sections and stained with markers for tumor-infiltrating lymphocytes (TILs; CD3, CD8) and immune checkpoint proteins (PD-1, PD-L1). Staining was performed using a Leica Bond RX Automated Stainer (Leica Products/Equipment, Leica Microsystems, Inc., Buffalo Groove, IL). Slides with incubated for 30 minutes at 95°C, followed by dewaxing with Leica Bond Dewax solution (Leica Biosystems, Inc., Buffalo Groove, IL; Cat no. AR9222), antigen retrieval with Bond Epitope Retrieval 1 solution (Leica Biosystems, Inc., Buffalo Groove, IL; Cat no. AR9961) and blocking of unspecific binding sites with 2% goat serum. Primary antibody binding was visualized with diaminobenzidine chromogen and a hematoxylin counterstain, using the Leica Bond Refine Detection kit (Leica Biosystems, Inc., Buffalo Groove, IL; Cat no. DS9800). Primary antibodies were diluted in Leica Bond Antibody Diluent buffer (Leica Biosystems, Inc., Buffalo Groove, IL; Cat no. AR9352) as follows: anti-CD3 (Abcam, Cambridge, MA, USA; Cat no. ab5690; rabbit polyclonal) 1:300, anti-CD8 (Abcam, Cambridge, MA, USA; Cat no. ab4055; rabbit polyclonal) 1:300), anti-PD-1 (Abcam, Cambridge, MA, USA; Cat no. ab137132; rabbit polyclonal) 1:75, anti-PD-L1 (Abcam, Cambridge, MA, USA; Cat no. ab205921; rabbit polyclonal) 1:100).

### 4.3. Analogue Quantification of T Cell Density and Immune Checkpoint Protein Expression

Analogue quantification of protein expression (CD3, CD8, PD-1, PD-L1) in the central tumor (CT) and in the invasive margin (IM) was performed by two independent trained investigators in a double-blinded manner. T cell density was defined as no (0), low (1), moderate (2) or high (3) with respect to positive T cells or TILs present. When different results were determined by the 2 independent investigators, samples were re-evaluated together and an agreed final score was determined.

### 4.4. Virtual Tissue Microarray Establishment

For comparison of quantitative marker expression, full tissue sections were scanned and images were acquired with an Axio Imager Z2 microscope, using a PixeLINK PL-D674 CU Camera and a 20x objective (with a 0.5 Aperture). Virtual TMA was established by randomly selecting two virtual cores each in the CT and IM using StrataQuestTM^®^ (TissueGnostics, Vienna, Austria). Digital analysis was performed on the acquired images. The Color Separation engine was used on the original BF RGB images to generate two grey scale images for the blue hematoxylin marker and the brown T cell marker. The blue shade image was used for automatic cell detection. Measurements (for CD3 and CD8 positive T cells) were generated using the Global Standard Measurements engine. Finally, manual correction was performed to correct automatic cell detection as well as to determine whether the manual marker assessment was positive or negative. Statistics were generated automatically based on total event count, total valid marker count, count and mean intensity of brown-positive cells as well as count and mean intensity of brown-negative cells; results were then corrected for manually-validated positive or negative cells. The process of digital analysis, from automated cell detection via marker assessment and manual correction to the final results of positive and negative T cells is shown in Supplementary Figure S2.

### 4.5. Immunoscore Calculation

TMA core sizes (CT - 0.6 mm diameter; IM - 1 mm diameter) and the cut-off values for T cell density for Immunoscore calculation in RC were chosen as previously proposed [18]. In short, each tumor was categorized into high or low density for CD3 and CD8 expression (n = cells/mm^2^) in each tumor area according to cut-off values predetermined using the minimum *p* value approach (CD3 CT, 256 cells/mm^2^; CD3 IM, 144 cells/mm^2^; CD8 CT, 202 cells/mm^2^; CD8 IM, 50 cells/mm^2^). Patient stratification was performed according to a ‘four-category score’ based on the respective cut-offs (i.e., 0-4) or a ‘dichotomized score’ based on the 0–4 score, i.e., 0 versus 1–4.

### 4.6. Estimation of Approximation of T Cell Density Quantified by Regions of Interest vs. Full Tissue Sections

In order to estimate the tissue area needed to approximate the T cell densities quantified on virtual TMA to the T cell densities on full sections, another 4 additional cores were randomly added to each area (CT, IM). Hence, a total of 6 cores per area were analyzed, which represented the maximum possible due to the size of tumor tissue available. Digital quantification was performed for CD3 and CD8 T cell densities as described above.

### 4.7. Statistical Analysis

Statistical analyses were performed in R v3.6.0 (The R Foundation for Statistical Computing, Vienna, Austria [41]. Kaplan Meier estimates and Cox regressions for overall survival were conducted with the functions survfit and coxph of the R-package survival v2.44–1.1 [42]. Final Cox models were selected by the backward selection method minimizing the Akaike Information Criterion (AIC) with function stepAIC of R-package MASS v7.3–51.4 [43] Correlations between analogue whole slide and digitally determined core marker expression values were calculated with the Spearman’s rank correlation coefficient method. Differences in marker expression values between the groups were estimated using the non-parametric Kruskal Wallis test.

## 5. Conclusion

We conclude that the tissue area used for immune marker quantification represents a crucial factor for the evaluation of prognostic biomarker potential. An option to avoid the above-mentioned pitfalls was presented in a recent study that demonstrated an optimized digital method using whole slide imaging for investigation of the spatial quantitative expression of immune cells in CRC samples by virtual microscopy [32,44]. The international validation of the Immunoscore was based on a respective image-analysis system with digital pathology software [31]. By using such methods, it can be assumed that quantification bias is kept to a minimum [45,46]; at the same time, digital processing is effective and tissue-saving.

## Figures and Tables

**Table 1:** Patient and tumor characteristics in relation to the level of CD3 T cell expression determined analogously on full tissue sections.

		CD3 – central tumor				CD3 – invasive margin			
Factor	No. of patients (%)	0	1	2	3	0	1	2	3
	**75 (100.0%)**	9 (12.0%)	34 (45.7%)	19 (25.3%)	13 (17.3%)	8 (10.7%)	28 (37.3%)	28 (37.3%)	1 (14.7%)
**Median age (range)**	**77 (51–94)**	n.s.*				n.s.*			
**Sex**		n.s.**				n.s.**			
**female**	**31 (41.3%)**	3 (4.0%)	16 (21.3%)	7 (9.3%)	5 (6.7%)	3 (4.0%)	12 (16.0%)	13 (17.3%)	3 (4.0%)
**male**	**44 (58.7%)**	6 (8.0%)	18 (24.0%)	12 (16.0%)	8 (19.7%)	5 (6.7%)	16 (21.3%)	15 (20.0%)	8 (10.7%)
**Preoperative RTx**		n.s.**				*p =* 0.006**			
**Yes**	**60 (80.0%)**	26 (34.7%)	9 (12.0%)	26 (34.7%)	9 (12.0%)	26 (34.7%)	9 (12.0%)	26 (34.7%)	3 (4.0%)
**No**	**15 (20.0%)**	8 (10.7%)	0 (0.0%)	8 (10.7%)	0 (0.0%)	8 (10.7%)	0 (0.0%)	8 (10.7%)	4 (5.3%)
**Tumor grading (G)**		n.s.*				n.s.*			
**Good (G1)**	**1 (1.3%)**	0 (0.0%)	0 (0.0%)	1 (1.3%)	0 (0.0%)	0 (0.0%)	0 (0.0%)	1 (1.3%)	0 (0.0%)
**Moderate (G2)**	**63 (84.0%)**	8 (10.7%)	25 (37.3%)	15 (20.0%)	12 (16.0%)	8 (10.7%)	22 (29.3%)	25 (33.3%)	8 (10.7%)
**Poor (G3)**	**11 (14.7%)**	1 (1.3%)	6 (8.0%)	3 (4.0%)	1 (1.3%)	0 (0.0%)	6 (8.0%)	2 (2.7%)	3 (4.0%)
**Dukes stage**		n.s.*				n.s.*			
**Stage A**	**21 (28.0%)**	2 (2.7%)	7 (9.3%)	6 (8.0%)	6 (8.0%)	2 (2.7%)	6 (8.0%)	12 (16.0%)	1 (1.3%)
**Stage B**	**25 (33.3%)**	3 (4.0%)	14 (18.7%)	4 (5.3%)	4 (5.3%)	2 (2.7%)	11 (14.7%)	9 (12.0%)	3 (4.0%)
**Stage C**	**24 (32.0%)**	2 (2.7%)	12 (16.0%)	8 (10.7%)	2 (2.7%)	3 (4.0%)	11 (14.7%)	3 (4.0%)	7 (9.3%)
**Stage D**	**5 (6.7%)**	2 (2.6%)	1 (1.3%)	1 (1.3%)	1 (1.3%)	1 (1.3%)	0 (0.0%)	4 (5.3%)	0 (0.0%)

**Note.** RTx: radiation therapy (hyper-fractionated short-term radiation protocol; 2× 2.5 Gy/d during 5d: total dose of 25 Gy); RC: rectal cancer; progressive disease: lymph node and/or distant metastases; *Spearman correlation statistical significance, **Approximative Linear-by-Linear Association Test; bold *p*-values—statistical significance at the *p* < 0.05 level.

**Table 2:** Patient and tumor characteristics in relation to the level of CD8 T cell expression determined analogously on full tissue sections.

		CD8 – central tumor				CD8 – invasive margin			
Factor	No. of patients (%)	0	1	2	3	0	1	2	3
	**75 (100.0%)**	15 (20.0%)	46 (61.3%)	10 (13.3%)	4 (5.3%)	12 (16.0%)	37 (49.3%)	19 (25.3%)	7 (9.3%)
**Median age (range)**	**77 (51–94)**	n.s.*				n.s.*			
**Sex**		n.s.**				n.s.**			
**female**	**31 (41.3%)**	7 (9.3%)	19 (25.3%)	3 (4.0%)	2 (2.7%)	4 (5.3%)	17 (22.7%)	7 (9.3%)	3 (4.0%)
**male**	**44 (58.7%)**	8 (10.7%)	27 (36.0%)	7 (9.3%)	2 (2.7%)	8 (10.7%)	20 (26.7%)	12 (16.0%)	4 (5.3%)
**Preoperative RTx**		*p =* 0.017**				*p =* 0.001**			
**Yes**	**60 (80.0%)**	15 (20.0%)	36 (48.0%)	7 (9.3%)	2 (2.7%)	12 (16.0%)	35 (46.7%)	10 (13.3%)	3 (4.0%)
**No**	**15 (20.0%)**	0 (0.0%)	10 (13.3%)	3 (4.0%)	2 (2.7%)	0 (0.0%)	2 (2.7%)	9 (12.0%)	4 (5.3%)
**Tumor grading (G)**		n.s.*				n.s.*			
**Good (G1)**	**1 (1.3%)**	0 (0.0%)	1 (1.3%)	0 (0.0%)	0 (0.0%)	0 (0.0%)	1 (1.3%)	0 (0.0%)	0 (0.0%)
**Moderate (G2)**	**63 (84.0%)**	13 (17.3%)	37 (49.3%)	10 (13.3%)	3 4.0%)	10 (13.3%)	30 (40.0%)	17 (22.7%)	6 (8.0%)
**Poor (G3)**	**11 (14.7%)**	2 (2.7%)	8 (10.7%)	0 (0.0%)	1 (1.3%)	2 (2.7%)	6 (8.0%)	2 (2.7%)	1 (1.3%)
**Dukes stage**		n.s.*				n.s.*			
**Stage A**	**21 (28.0%)**	4 (5.3%)	11 (14.7%)	6 (8.0%)	0 (0.0%)	2 (2.7%)	12 (16.0%)	7 (9.3%)	0 (0.0%)
**Stage B**	**25 (33.3%)**	6 (8.0%)	15 (20.0%)	2 (2.7%)	2 (2.7%)	5 (6.7%)	12 (16.0%)	5 (6.7%)	3 (4.0%)
**Stage C**	**24 (32.0%)**	4 (5.3%)	17 (22.7%)	1 (1.3%)	2 (2.7%)	5 (6.7%)	11 (14.7%)	5 (6.7%)	3 (4.0%)
**Stage D**	**5 (6.7%)**	1 (2.7%)	3 (4.0%)	1 (2.7%)	0 (0.0%)	0 (0.0%)	2 (2.7%)	2 (2.7%)	1 (1.3%)

**Note.** RTx: radiation therapy (hyper-fractionated short-term radiation protocol; 2× 2.5 Gy/d during 5d: total dose of 25 Gy); RC: rectal cancer; progressive disease: lymph node and/or distant metastases; *Spearman correlation statistical significance, **Approximative Linear-by-Linear Association Test; bold *p*-values—statistical significance at the *p* < 0.05 level.

**Table 3:** Patient and tumor characteristics in relation to the level of PD-1 immune checkpoint protein expression determined analogously on full tissue sections.

		PD-1 – central tumor*			PD-1 – invasive margin*		
Factor	No. of patients (%)	0	1	2	0	1	2
	**75 (100%)**	25 (33.3%)	42 (56.0%)	8 (10.7%)	26 (34.7%)	37 (49.3%)	12 (16.0%)
**Median age (range)**	**77 (51–94)**	n.s.**			n.s.**		
**Sex**		n.s.***			n.s.***		
**female**	**31 (41.3%)**	10 (13.3%)	19 (25.3%)	2 (2.7%)	12 (16.0%)	13 (17.3%)	6 (8.0%)
**male**	**44 (58.7%)**	15 (20.0%)	23 (30.7%)	6 (8.0%)	14 (18.7%)	24 (32.0%)	6 (8.0%)
**Preoperative RTx**		n.s.***			*p =* 0.005***		
**Yes**	**60 (80.0%)**	23 (30.7%)	31 (41.3%)	6 (8.0%)	2 (2.7%)	7 (9.3%)	6 (8.0%)
**No**	**15 (20.0%)**	2 (2.7%)	11 (14.6%)	2 (2.7%)	24 (32.0%)	30 (40.0%)	6 (8.0%)
**Tumor grading (G)**		n.s.**			n.s.**		
**Good (G1)**	**1 (1.3%)**	0 (0.0%)	1 (1.3%)	0 (0.0%)	0 (0.0%)	1 (1.3%)	0 (0.0%)
**Moderate (G2)**	**63 (84.0%)**	21 (28.0%)	35 (46.7%)	7 (9.3%)	22 (29.3%)	31 (41.3%)	10 (13.3%)
**Poor (G3)**	**11 (14.7%)**	4 (5.3%)	6 (8.0%)	1 (1.3%)	4 (5.3%)	5 (6.7%)	2 (2.7%)
**Dukes stage**		n.s.**			n.s.**		
**Stage A**	**21 (28.0%)**	4 (5.3%)	13 (17.4%)	4 (5.3%)	3 (4.0%)	15 (20.0%)	3 (4.0%)
**Stage B**	**25 (33.3%)**	11 (14.7%)	11 (14.6%)	3 (4.0%)	11 (14.6%)	10 (13.3%)	4 (5.3%)
**Stage C**	**24 (32.0%)**	9 (12.0%)	14 (18.7%)	1 (1.3%)	11 (14.6%)	10 (13.3%)	3 (4.0%)
**Stage D**	**5 (6.7%)**	1 (1.3%)	4 (5.3%)	0 (0.0%)	1 (1.3%)	2 (2.7%)	2 (2.7%)

**Note.** *PD-1 expression was exclusively found on tumor infiltrating lymphocytes. RTx: radiation therapy (hyper-fractionated short-term radiation protocol; 2× 2.5 Gy/d during 5d: total dose of 25 Gy); RC: rectal cancer; progressive disease: lymph node and/or distant metastases; **Spearman correlation statistical significance, ***Approximative Linear-by-Linear Association Test; bold *p*-values—statistical significance at the *p* < 0.05 level.

**Table 4:** Patient and tumor characteristics in relation to the level of PD-L1 immune checkpoint protein expression determined analogously on full tissue sections.

		PD-L1 – central tumor*				PD-L1 – invasive margin*			
Factor	No. of patients (%)	0	1	2	3	0	1	2	3
	**75 (100.0%)**	42 (56.0%)	15 (20.0%)	15 (20.0%)	3 (4.0%)	47 (62.7%)	12 (16.0%)	13 (17.3%)	3 (4.0%)
**Median age (range)**	**77 (51–94)**	n.s.**				n.s.**			
**Sex**		n.s.***				n.s.***			
**female**	**31 (41.3%)**	16 (21.3%)	7 (9.3%)	6 (8.0%)	2 (2.7%)	20 (26.7%)	4 (5.3%)	6 (8.0%)	1 (1.3%)
**male**	**44 (58.7%)**	26 (34.7%)	8 (10.7%)	9 (12.0%)	1 (1.3%)	27 (36%)	8 (10.7%)	7 (9.3%)	2 (2.7%)
**Preoperative RTx**		n.s.***				n.s.***			
**Yes**	**60 (80.0%)**	36 (48.0%)	10 (13.3%)	11 (14.7%)	3 (4.0%)	40 (53.3%)	9 (12.0%)	8 (10.7%)	3 (4.0%)
**No**	**15 (20.0%)**	6 (8.0%)	5 (6.7%)	4 (5.3%)	0 (0.0%)	7 (9.3%)	3 (4.0%)	5 (6.7%)	0 (0.0%)
**Tumor grading (G)**		n.s.**				n.s.**			
**Good (G1)**	**1 (1.3%)**	0 (0.0%)	0 (0.0%)	1 (1.3%)	0 (0.0%)	0 (0.0%)	0 (0.0%)	1 (1.33%)	0 (0.0%)
**Moderate (G2)**	**63 (84.0%)**	36 (48.0%)	14 (18.7%)	10 (13.3%)	3 (4.0%)	39 (52.0%)	11 (14.7%)	10 (13.3%)	3 (4.0%)
**Poor (G3)**	**11 (14.7%)**	6 (8.0%)	1 (1.33%)	4 (5.3%)	0 (0.0%)	8 (10.7%)	1 (1.3%)	2 (3.7%)	0 (0.0%)
**Dukes stage**		R = −0.23, *p =* 0.046**				R *= −*0.24, *p =* 0.041**			
**Stage A**	**21 (28.0%)**	10 (13.3%)	3 (4.0%)	7 (9.3%)	1 (1.3%)	9 (12.0%)	4 (5.3%)	6 (8.0%)	2 (3.7%)
**Stage B**	**25 (33.3%)**	12 (16.0%)	7 (9.3%)	4 (5.3%)	2 (2.7%)	18 (24.0%)	3 (4.0%)	3 (4.0%)	1 (1.3%)
**Stage C**	**24 (32.0%)**	16 (21.3%)	4 (5.3%)	4 (5.3%)	0 (0.0%)	16 (21.2%)	4 (5.4%)	4 (5.4%)	0 (0.0%)
**Stage D**	**5 (6.7%)**	4 (5.3%)	1 (1.3%)	0 (0.0%)	0 (0.0%)	4 (5.3%)	1 (1.3%)	0 (0.0%)	0 (0.0%)

**Note.** *PD-L1 expression was exclusively found on tumor infiltrating lymphocytes. RTx: radiation therapy (hyper-fractionated short-term radiation protocol; 2× 2.5 Gy/d during 5d: total dose of 25 Gy); RC: rectal cancer; progressive disease: lymph node and/or distant metastases; **Spearman correlation statistical significance, ***Approximative Linear-by-Linear Association Test, bold *p*-values—statistical significance at the *p* < 0.05 level.

**Table 5:** Univariate (**A**) and multiple Cox Regression analysis (**B**) for disease-free survival of rectal cancer patients quantified using analogue quantification for T cell densities (full sections).

**Table A**	**Univariate Analysis**		
**Factor**	**HR**	**CI_95_**	***p*-value**
**CD3 (CT)**	0.45	0.23–0.88	**0.018**
**CD3 (IM)**	0.33	0.16–0.65	**0.002**
**CD8 (CT)**	n.s.		
**CD8 (IM)**	0.48	0.23.1.00	**0.049**
**PD-1 (CT)**	0.36	0.14–0.94	**0.037**
**PD-1 (IM)**	0.30	0.11–0.79	**0.016**
**PD-L1 (CT)**	0.51	0.24–1.10	0.087
**PD-L1 (IM)**	0.42	0.16–1.10	0.078
**Radiation therapy (25 Gy)**	n.s.		
**Nodal positive RC**	3.69	1.23–11.08	**0.020**
**Table B**	**Multiple Model**		
**Factor**	**HR**	**CI_95_**	***p*-value**
CD3 (CT)*	n.s.		
**Nodal positive RC**	2.42	0.10–5.87	0.050
CD3 (IM)*	0.46	0.27–0.79	**0.004**
**Nodal positive RC**	2.49	1.03–6.05	**0.043**
CD8 (CT)*	n.s.		
**Nodal positive RC**	2.69	1.12–6.53	**0.029**
CD8 (IM)*	0.59	0.33–1.05	0.074
**Nodal positive RC**	2.78	1.14–6.74	**0.024**
PD-1 (CT)*	0.45	0.20–0.99	**0.048**
**Nodal positive RC**	2.45	1.01–5.94	**0.047**
PD-1 (IM)*	0.48	0.23–0.98	**0.045**
**Nodal positive RC**	2.50	1.03–6.07	**0.043**
PD-L1 (CT)*	0.54	0.28–1.04	0.067
**Nodal positive RC**	2.13	0.87–5.21	0.099
PD-L1 (IM)*	0.47	0.22–1.00	0.050
**Nodal positive RC**	2.38	0.98–5.76	0.056

**Note.** Table A – univariate analysis; Table B – multiple model: double lines indicate separate models; CT: central tumor, IM: invasive margin; RC: rectal cancer; HR: hazard ratio, CI: confidence interval; radiation therapy according to a hyper-fractionated short-term radiation protocol (preoperative administration of 2× 2.5 Gy/d during 5d: total dose of 25 Gy); PD-1 and PD-L1 expression was found on leucocytes; n.s.: not significant; *radiation therapy (25 Gy) was excluded from the model by the backward elimination procedure minimizing the Akaike information criterion (AIC); bold *p*-values—statistical significance at the *p* < 0.05 level.

**Table 6:** Univariate and multiple Cox Regression analysis for overall survival of rectal cancer patients quantified using analogue quantification for TC densities (full sections).

**Table A**	**Univariate Analysis**		
**Factor**	**HR**	**CI_95_**	***p*-value**
**CD3 (CT)**	0.45	0.23–0.88	**0.018**
**CD3 (IM)**	0.33	0.16–0.65	**0.002**
**CD8 (CT)**	n.s.		
**CD8 (IM)**	0.48	0.23–1.00	**0.049**
**PD-1 (CT)**	0.36	0.14–0.94	**0.037**
**PD-1 (IM)**	0.30	0.11–0.79	**0.016**
**PD-L1 (CT)**	0.51	0.24–1.10	0.087
**PD-L1 (IM)**	0.42	0.16–1.10	0.078
**Radiation therapy (25 Gy)**	n.s.		
**Nodal positive RC**	3.69	1.23–11.08	**0.020**
**Table B**	**Multiple Models**		
**Factor**	**HR**	**CI_95_**	***p*-value**
CD3 (CT)*	0.46	0.23–0.89	**0.021**
**Nodal positive RC**	3.51	1.17–10.53	0.025
CD3 (IM)*	0.35	0.18–0.69	**0.002**
**Nodal positive RC**	3.44	1.14–10.33	**0.028**
CD8 (CT)*	0.44	0.21–0.92	**0.029**
**Nodal positive RC**	4.18	1.39–12.57	0.011
CD8 (IM)*	0.44	0.21–0.92	**0.029**
**Nodal positive RC**	4.18	1.39–12.57	0.011
PD-1 (CT)*	0.35	0.13–0.94	**0.037**
**Nodal positive RC**	3.62	1.21–10.87	**0.022**
PD-1 (IM)*	0.30	0.11–0.81	**0.017**
**Nodal positive RC**	3.60	1.20–10.86	**0.023**
**PD-L1 (CT)**	n.i.		
**Nodal positive RC**	4.26	1.41–12.86	**0.010**
**Radiation Therapy (25Gy)**	n.s.		
PD-L1 (IM)*	0.44	0.16–1.17	0.099
**Nodal positive RC**	3.36	1.12–10.10	**0.031**

**Note.** Table A – univariate analysis; Table B – multiple model: double lines indicate separate models; CT: central tumor, IM: invasive margin; RC: rectal cancer; HR: hazard ratio, CI: confidence interval; radiation therapy according to hyper-fractionated short-term radiation protocol (preoperative administration of 2× 2.5 Gy/d during 5d: total dose of 25 Gy); PD-1 and PD-L1 expression was found on leucocytes; n.s.: not significant; n.i.: not included/*radiation therapy (25 Gy) was excluded from the model by the backward elimination procedure minimizing the Akaike information criterion (AIC) ; bold *p*-values – statistical significance at the *p* < 0.05 level.

**Table 7:** Univariate and multiple Cox Regression analysis for disease-free survival of rectal cancer patients using the Immunoscore (2 cores per tumor area, virtual TMA).

Factor	Univariate Analysis*			Multiple Model*		
	HR	CI_95_	*p*-value	HR	CI_95_	*p*-value
**Immunoscore**	n.s.			n.s.		
**Radiation therapy (25 Gy)**	n.s.			n.s.		
**Nodal positive RC**	n.s.			n.s.		

**Note.** Radiation therapy according to a hyper-fractionated short-term radiation protocol (preoperative administration of 2× 2.5 Gy/d during 5d: total dose of 25 Gy); RC: rectal cancer; n.s.: not significant; n.i.: not included: excluded from the model by the backward elimination procedure minimizing the Akaike information criterion (AIC); *no differences were found in whether survival analysis was performed across all groups or compared between the radiation therapy group and the treatment-naïve group.

**Table 8:** Univariate and multiple Cox Regression analysis for overall survival of rectal cancer patients using the Immunoscore (2 cores per tumor area, virtual TMA).

Factor	Univariate Analysis*			Multiple Model*		
	HR	CI_95_	*p*-value	HR	CI_95_	*p*-value
**Immunoscore**	n.s.			n.i.		
**Radiation therapy (25 Gy)**	n.s.			n.i.		
**Nodal positive RC**	n.s.			n.s.		

**Note.** Radiation therapy according to a hyper-fractionated short-term radiation protocol (preoperative administration of 2× 2.5 Gy/d during 5d: total dose of 25 Gy); RC: rectal cancer; n.s.: not significant; n.i.: not included: excluded from the model by the backward elimination procedure minimizing the Akaike information criterion (AIC); *no differences were found in whether survival analysis was performed across all groups or compared between the radiation therapy group and the treatment-naïve group.

**Table 9:** Univariate and multiple Cox Regression analysis for disease-free survival of rectal cancer patients (6 cores per tumor region, virtual TMA).

**Table A**	**Univariate Analysis**		
**Factor**	**HR**	**CI_95_**	***p*-value**
**CD3 (CT)**	0.38	0.10–1.20	0.093
**CD3 (IM)**	n.s.		
**CD8 (CT)**	n.s.		
**CD8 (IM)**	n.s.		
**Radiation therapy (25 Gy)**	n.s.		
**Nodal positive RC**	n.s.		
**Table B**	**Multiple Model**		
**Factor**	**HR**	**CI_95_**	***p*-value**
CD3 (CT)*	0.34	0.10-1.20	0.093
**Nodal positive RC**	n.i.		
CD3 (IM)*	n.i.		
**Nodal positive RC**	n.s.		
CD8 (CT)*	n.i.		
**Nodal positive RC**	n.s.		
CD8 (IM)*	n.i.		
**Nodal positive RC**	n.s.		

**Note.** Table A – univariate analysis; Table B – multiple model: double lines indicate separate models; CT: central tumor, IM: invasive margin; HR: hazard ratio, CI: confidence interval; radiation therapy according to a hyper-fractionated short-term radiation protocol (preoperative administration of 2× 2.5 Gy/d during 5d: total dose of 25 Gy); RC: rectal cancer; n.s.: not significant; n.i.: not included/*radiation therapy (25 Gy) was excluded from the model by the backward elimination procedure minimizing the Akaike information criterion (AIC); trend towards statistical significance at the *p* < 0.1 level.

**Table 10:** Univariate and multiple Cox Regression analysis for overall survival of rectal cancer patients (6 cores per tumor region, virtual TMA).

**Table A**	**Univariate Analysis**		
**Factor**	**HR**	**CI_95_**	***p*-value**
**CD3 (CT)**	0.20	0.04-1.01	0.052
**CD3 (IM)**	n.s.		
**CD8 (CT)**	n.s.		
**CD8 (IM)**	n.s.		
**Radiation therapy (25 Gy)**	n.s.		
**Nodal positive RC**	2.88	0.90–9.20	0.074
**Table B**	**Multiple Model**		
**Factor**	**HR**	**CI_95_**	***p*-value**
CD3 (CT)*	0.23	0.04–1.22	0.084
**Nodal positive RC**	n.s.		
CD3 (IM)*	n.i.		
**Nodal positive RC**	2.88	0.90–9.20	0.074
CD8 (CT)*	n.i.		
**Nodal positive RC**	2.88	0.90–9.20	0.074
CD8 (IM)*	n.i.		
**Nodal positive RC**	2.88	0.90–9.20	0.074

**Note.** Table A – univariate analysis; Table B – multiple model: double lines indicate separate models; CT: central tumor, IM: invasive margin; HR: hazard ratio, CI: confidence interval; radiation therapy according to a hyper-fractionated short-term radiation protocol (preoperative administration of 2× 2.5 Gy/d during 5d: total dose of 25 Gy); RC: rectal cancer; n.s.: not significant; n.i.: not included/*radiation therapy (25 Gy) was excluded from the model by the backward elimination procedure minimizing the Akaike information criterion (AIC); trend towards statistical significance at the *p* < 0.1 level.

## Supplementary Materials

The following are available online at https://www.mdpi.com/2072-6694/12/3/563/s1, Figure S1: T cell density (CD3, CD8) and immune checkpoint protein expression (PD-1, PD-L1) in rectal cancer samples exemplifying how real tissue microarray cores miss immune cell clusters. Figure S2: Process of digital analysis of CD8 marker expression using StrataQuestTM^®^.
