# Supplementary figures and images for: The Determination of Immunomodulation and Its Impact on Survival of Rectal Cancer Patients Depends on the Area Comprising a Tissue Microarray
Source: Cancers (Basel). 2020 Feb 29;12(3):563. doi: 10.3390/cancers12030563 (PMC7139832; doi:10.3390/cancers12030563)

**Supplementary Fig. 1**

**CD3**

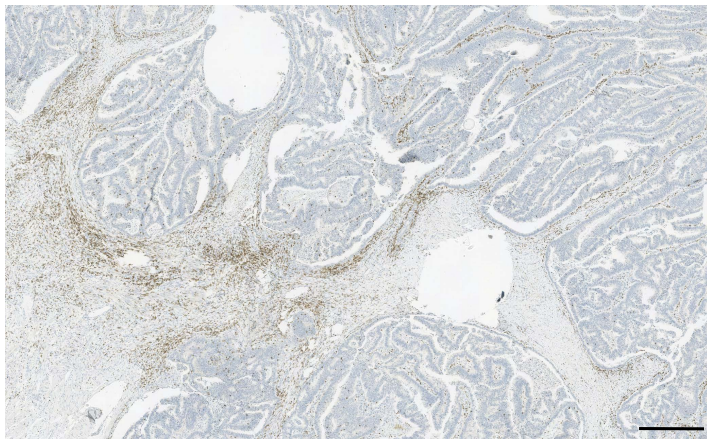

**CD8**

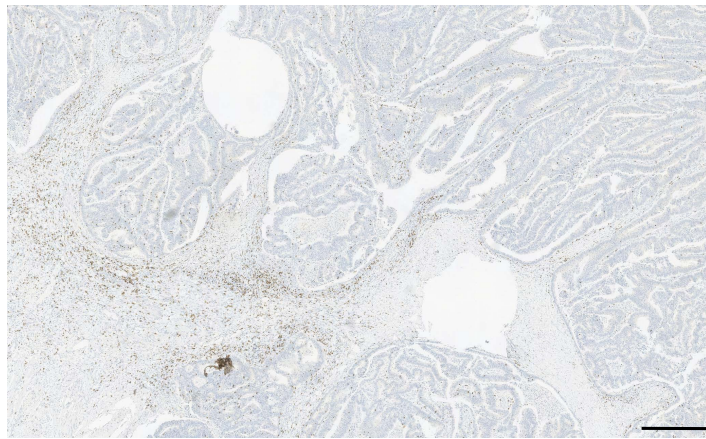

**PD-1**

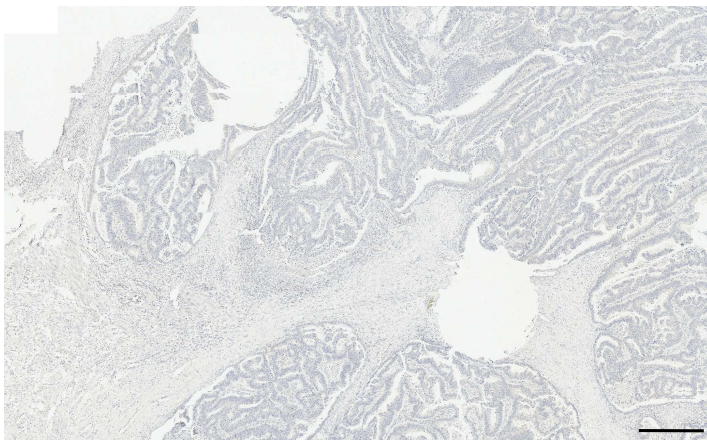

**PD-L1**

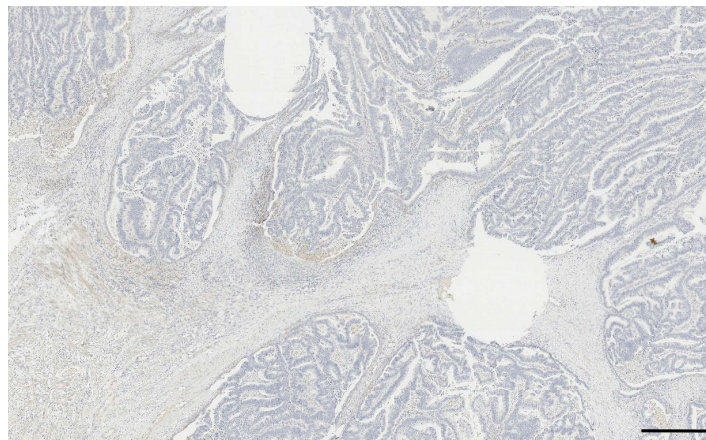

Supplementary Fig. 2

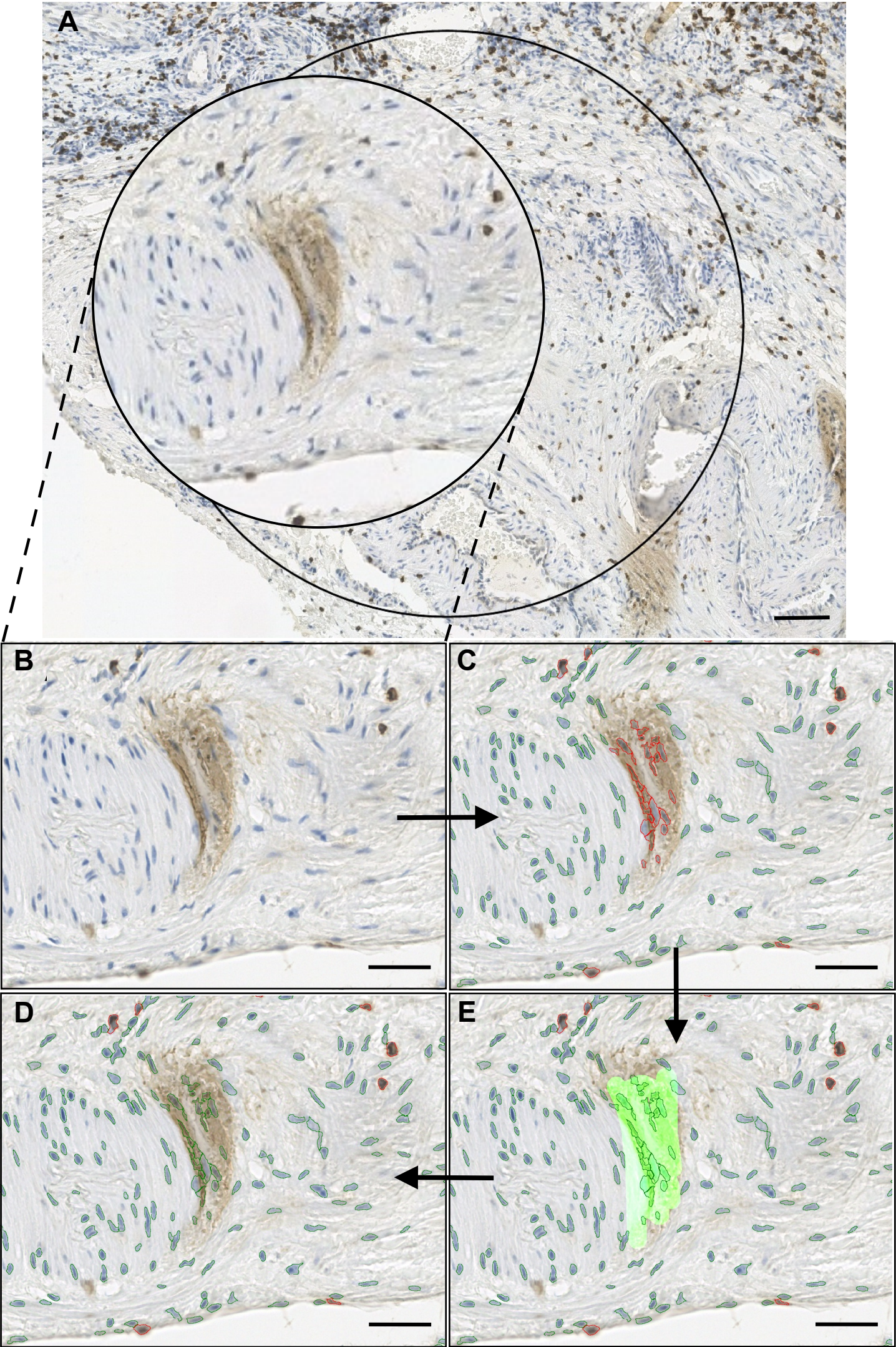

Supplement: Supplementary file 1 [file cancers-12-00563-s001.pdf]
